# Household air pollution, chronic respiratory disease and pneumonia in Malawian adults: A case-control study

**DOI:** 10.12688/wellcomeopenres.12621.1

**Published:** 2017-10-24

**Authors:** Hannah R. Jary, Stephen Aston, Antonia Ho, Emanuele Giorgi, Newton Kalata, Mulinda Nyirenda, Jane Mallewa, Ingrid Peterson, Stephen B. Gordon, Kevin Mortimer

**Affiliations:** 1 Malawi-Liverpool-Wellcome Trust Clinical Research Programme, Chichiri, Blantyre 3, Malawi; 2 Liverpool School of Tropical of Medicine, Liverpool, L3 5QA, UK; 3 Tropical and Infectious Diseases Unit, Royal Liverpool University Hospital, Liverpool, L7 8XP, UK; 4 Institute of Infection and Global Health, University of Liverpool, Liverpool, L69 7BE, UK; 5 Lancaster Medical School, Lancaster University, Lancaster, LA1 4YW, UK; 6 College of Medicine, University of Malawi, Chichiri, Blantyre 3, Malawi; 7 Queen Elizabeth Central Hospital, Chichiri, Blantyre 3, Malawi

**Keywords:** Household air pollution, Pneumonia, Chronic respiratory disease, Particulate matter, Carbon monoxide, Malawi, Sub-Saharan Africa

## Abstract

**Background: **Four million people die each year from diseases caused by exposure to household air pollution. There is an association between exposure to household air pollution and pneumonia in children (half a million attributable deaths a year); however, whether this is true in adults is unknown. We conducted a case-control study in urban Malawi to examine the association between exposure to household air pollution and pneumonia in adults.

**Methods: **Hospitalized patients with radiologically confirmed pneumonia (cases) and healthy community controls underwent 48 hours of ambulatory and household particulate matter (µg/m ^3^) and carbon monoxide (ppm) exposure monitoring. Multivariate logistic regression, stratified by HIV status, explored associations between these and other potential risk factors with pneumonia.

**Results: **145 (117 HIV-positive; 28 HIV-negative) cases and 253 (169 HIV-positive; 84 HIV-negative) controls completed follow up. We found no evidence of association between household air pollution exposure and pneumonia in HIV-positive (e.g. ambulatory particulate matter adjusted odds ratio [aOR] 1.00 [95% CI 1.00–1.01, p=0.141]) or HIV-negative (e.g. ambulatory particulate matter aOR 1.00 [95% CI 0.99–1.01, p=0.872]) participants. Chronic respiratory disease was associated with pneumonia in both HIV-positive (aOR 28.07 [95% CI 9.29–84.83, p<0.001]) and HIV-negative (aOR 104.27 [95% CI 12.86–852.35, p<0.001]) participants.

**Conclusions: **We found no evidence that exposure to household air pollution is associated with pneumonia in Malawian adults. In contrast, chronic respiratory disease was strongly associated with pneumonia.

## Introduction

Four million people die each year from diseases caused by exposure to household air pollution from the domestic burning of solid fuels ^1^. Half a million of these deaths are due to acute lower respiratory infections (ALRI) in young children ^2^. In adults, the majority of deaths are attributed to chronic obstructive lung disease, cardiovascular diseases, and lung cancer ^3^. Although plausible, it is not known if household air pollution is associated with ALRI in adults as it is in children ^4^.

In low-income areas such as Malawi, pneumonia is the commonest cause of admission to hospital for adults and has a high fatality rate ^5– 7^. HIV infection is a well-established risk factor for pneumonia; the extent to which other factors, such as household air pollution and other poverty-related exposures, affect the risk of pneumonia has not been adequately studied ^4, 8^. In low-income countries burdened with high rates of adult pneumonia, domestic use of solid fuel is widespread ^1^. If an association between household air pollution and adult ALRI is found, the attributable risk is potentially high.

We conducted a case-control study, The Acute Infection of the Respiratory tract (AIR) study, to test the hypothesis that household air pollution and chronic respiratory disease (CRD) are associated with an increased risk of pneumonia in adults living in urban Malawi.

## Methods

### Setting

Malawi, population 16.7 million, is one of the world’s poorest countries, and has a life expectancy of 59 years ^9, 10^. Blantyre, Malawi’s second city, has a HIV prevalence of 18.5% ^11^. Queen Elizabeth Central Hospital (QECH) is a large government hospital providing free health care to a population of 1.3 million in greater Blantyre.

### Participants

Cases were defined by the presence of radiologically-confirmed pneumonia requiring hospitalisation and controls were defined by the absence of pneumonia. Inclusion and exclusion criteria are presented in Box 1.

**Box 1.** Inclusion and exclusion criteria for cases and controls.

	CASES	CONTROLS
**Inclusion criteria**	Age 18 years or over Resident in Blantyre city Reported cough or chest pain or breathlessness or hemoptysis Reported fever or recorded fever (≥38°C) Crepitations or pleural rub or bronchial breathing Radiological changes judged to be new and consistent with pneumonia, without another obvious cause Requires hospitalisation	Age 18 years or over Resident in Blantyre city
**Exclusion criteria**	Pre–admission diagnosis of terminal illness (e.g., metastatic malignancy, terminal AIDS) Current anti–tuberculosis treatment or evidence of current tuberculosis infection Prior hospitalisation within the last 4 weeks Prior participation in the study Lives in a residential institution (e.g., prison) Death prior to follow–up assessment Alternative diagnosis explaining their presentation Symptoms for 14 days or more	Pre–admission diagnosis of terminal illness (e.g., metastatic malignancy, terminal AIDS) Current anti–tuberculosis treatment or evidence of current tuberculosis infection Hospitalisation for a pneumonia–like illness in the past 4 months or current pneumonia–like illness Prior participation in the study Lives in a residential institution (e.g., prison) Death prior to follow–up assessment Utilizes private health care facilities if has illness requiring hospitalisation

**Note** For pragmatic reasons relating to resource availability, individuals could be recruited as a ‘provisional case’ prior to having a chest x-ray. Individuals were subsequently excluded if there was no evidence of pneumonia on chest x-ray or if they later met exclusion criteria (e.g. were commenced on tuberculosis treatment or died). Individuals were designated as a ‘case’ only when they had completed follow-up.

All adult medical admissions to QECH were screened for symptoms suggestive of pneumonia by study clinical officers to identify potential cases. For control recruitment, residential census enumeration areas were randomly selected from all enumeration areas within Blantyre city, with selection weighted by population size. Field workers followed randomly generated routes within these enumeration areas and screened all potential participants (including performing HIV tests) in each household along the route. A maximum of one individual was recruited per household, selected randomly. Screening continued until two controls had been recruited from that enumeration area. To supplement door–to–door recruitment, HIV–positive individuals attending community antiretroviral clinics within Blantyre city were also screened. Recruitment was stratified by HIV status to enable the data to be analyzed as two separate case–control studies. Within these two subgroups, controls were frequency–matched to cases by age (18–34 years or ≥35 years) and gender.

Cases and controls were contemporaneously recruited and followed-up throughout the study period, to account for temporal changes in air pollution exposure related to season. Case recruitment was from July 2014 until January 2016, and follow up appointments took place between September 2014 and March 2016. Control recruitment and follow up appointments took place from August 2014 until February 2016.

### Study procedures

Initial assessment of provisional cases included medical history and examination by study clinical officers, and diagnostic tests ( Box 2). Pneumonia was confirmed by chest X-ray review by a study clinical officer and a study doctor.

Box 2. **Hospital diagnostic tests for provisional cases.**

- HIV test +/- CD4 count
- Malaria rapid diagnostic test
- Blood culture
- BinaxNOW® *Streptococcus pneumoniae* urinary antigen
- Sputum for acid-fast bacilli smear, mycobacterial culture, and GeneXpert ^®^ MTB/RIF
- Pleural fluid specimen for acid-fast bacilli smear and mycobacterial culture (if clinically indicated)
- Chest X-ray

Follow-up assessments were conducted in the participants’ homes. Continuous ambulatory and household monitoring of particulate matter <2.5 µm diameter (PM _2.5_, µg/m ^3^) and carbon monoxide parts per million (CO, ppm) was performed for 48 hours. Participants wore backpacks with Aprovecho Indoor Air Pollution meters, while UCB-PATS (Particle and Temperature Sensor, University of California, Berkeley) and Lascar EL-USB-CO Data Logger monitors were placed 1 meter (m) from the household’s cooking stove or fire at an elevation of 1 m. Spirometry was conducted using an EasyOne Spirometer (ndd Medical Technologies, Zurich, Switzerland) to American Thoracic Society standards ^12^. NHANES III reference ranges, corrected for Caucasian ethnicity, were used to calculate predicted values. Two reviewers (HJ, and Lindsay Zurba (Spirometry Training Services Africa CC)) independently performed quality assurance and spirometry interpretation. Questionnaires (see Supplementary File 1), including items from the Burden of Obstructive Lung Disease (BOLD) questionnaires ^13^, evaluated a range of potential risk factors and socioeconomic status. The primary exposures of interest were mean ambulatory PM _2.5_ exposure and presence of CRD (defined using a composite questionnaire assessment ( Box 3)). The full study protocol has been published elsewhere ^14^.

Box 3. **Composite definition of chronic respiratory disease.**

Answering affirmative to any of the following in the BOLD questionnaire:

- Current usual cough
- Current usual sputum production
- Current breathlessness
- Wheeze in past 12 months
- Ever had diagnosis of emphysema
- Current diagnosis of chronic bronchitis
- Current diagnosis of asthma
- Current long-term respiratory medication

Note: Cases were asked to recall their status from 6 months previously, prior to their episode of pneumonia.

### Sample size

Based on assumptions of α=0.05, β=0.2, and an estimated percentage of controls with CRD of at least 15%, the target sample size was 160 cases and 160 controls in the HIV-positive subgroup (ratio 1:1) to detect an odds ratio (OR) of 2.2 or greater. A smaller exploratory study was planned with 60 cases and 90 controls in the HIV-negative subgroup (ratio 1:1.5).

### Statistical considerations

Data files were exported to Stata 13.1 (Statacorp, College Station, TX, USA) for analysis. Missing air pollution exposure data (for ambulatory PM _2.5_ and CO levels and household CO levels) were imputed using spatial interpolation. Due to the large number of missing data, household PM _2.5_ data were not imputed. Missing questionnaire data were imputed using multivariate multinomial models by exploiting their association with other observed variables. A socioeconomic status score was generated using principal components analysis, based on data regarding asset-based measures, education level, and household characteristics ^15^.

Univariate logistic regression, including *a priori* potential confounders, was performed for each subgroup. For analysis of the HIV-positive subgroup, multivariate forward stepwise logistic regression was performed for each of the main exposures of interest ( *a priori* potential confounders (as indicated in Table 2 and Supplementary File 2) were included in the model if their likelihood ratio test p-value was <0.2 (criteria for entry p<0.05 and removal p>0.1)). Adjustment in the HIV-negative subgroup was limited to frequency-matched factors (age and sex).

To test the hypothesis that pneumonia cases are spatially clustered, we used generalized additive models and smoothing latitude and longitude over the geographic reach of the study area ^16^.

### Ethical considerations

This study was approved by the College of Medicine Research Ethics Committee, University of Malawi (P.02/14/1518) and the Liverpool School of Tropical Medicine Research Ethics Committee (14.016). All participants gave written informed consent prior to participation in the study.

## Results

### Participant recruitment

**Figure 1.**
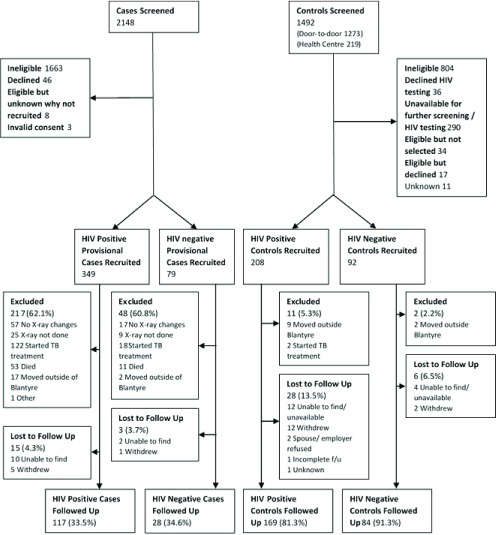
Participant flow chart showing the number of cases and controls screened, recruited, and followed up in the HIV–positive and HIV–negative subgroups.

**Figure 2.**
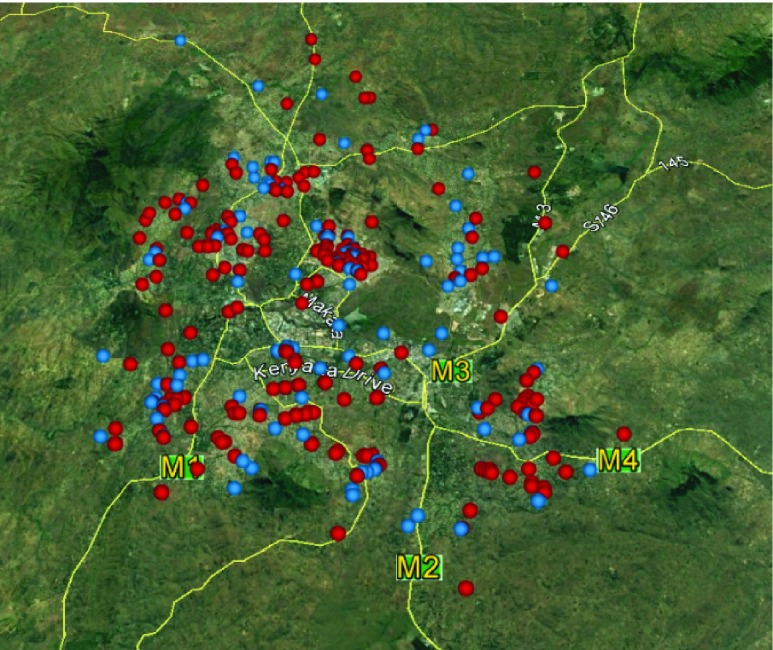
Participant household locations. A map of Blantyre city, Southern Region, Malawi showing the location of case ( •) and control ( •) households. Mapping software: Google Earth Pro (7.1.5.1157).

We screened 2148 and 1492 potential cases and controls, respectively, between July 2014 and February 2016. Of the screened cases, 58.5% were men with a median age of 36 years (interquartile range (IQR) 30–47). Of the screened controls, 61.6% were men, with a median age of 30 (IQR 23–40). From HIV-positive and HIV-negative groups, respectively, we recruited 349 and 79 provisional cases, and 208 and 92 controls ( Figure 1). Of the recruited participants, 64.7% and 59.3% were male, with a median age of 35 (IQR 30–42, range 18–89) and 35 (IQR 29–43, range 18–78) in the provisional cases and controls, respectively, and lived across Blantyre city ( Figure 2). The main reasons for ineligibility amongst potential screened cases were symptom duration greater than 14 days (913, 42.5%), lack of clinical signs consistent with pneumonia (416, 19.4%), living outside of urban Blantyre (385, 17.9%) and absence of fever (304, 14.2%). The main reasons for ineligibility amongst potential controls were not meeting HIV status/sex/age requirements for stratified recruitment (682, 45.7%), current evidence of tuberculosis or tuberculosis treatment (55, 3.7%), and recent pneumonia-like illness (45, 3.0%). One hundred and forty-five (117 HIV-positive, 28 HIV-negative) cases and 253 (169 HIV-positive, 84 HIV-negative) controls completed follow-up. Reasons for not completing follow-up amongst recruited provisional cases were subsequent exclusion for ineligibility (264, 61.7%; including lack of radiological evidence of pneumonia (93, 21.7%), commencement of tuberculosis treatment (114, 26.6%), and death (64, 15.0%)), and loss to follow-up (19, 4.4%) ( Figure 1). Among recruited controls, 34 individuals (11.3%) were lost to follow-up and 13 (4.3%) were ineligible and subsequently excluded.

### Baseline characteristics of cases

Comparisons between cases who completed follow-up and provisional cases who did not complete follow-up (predominantly due to ineligibility, see Figure 1) were made to explore evidence of potential selection bias ( Table 1). Those who completed follow-up had a higher admission CD4 count than those who were not followed up (median 119 cells/μl (IQR 47–205) vs. median 86 cells/μl (IQR 30–185)).

**Table 1.** Baseline hospital data for all recruited cases. Baseline clinical data for cases who completed follow up and provisional cases who did not complete follow up.

	Cases who completed follow-up (total n=145)	Provisional cases who did not complete follow-up (total n=283)
Symptom duration * (days), median (IQR)	7 (5–8)	7 (5–10)
Length of admission * (days), median (IQR)	5 (4–8)	7 (4–12)
Hospital outcome, n (%) Alive Dead Unknown	145 (100.0) 0 (0) 0 (0)	232 (82.0) 46 (16.3) 5 (1.8)
Pre-hospital antibiotics, n (%) Yes No Unknown	79 (54.5) 59 (40.7) 7 (4.8)	158 (55.8) 111 (39.2) 14 (4.9)
Systolic blood pressure * (mmHg), mean (STD)	104.4 (22.5)	112.1 (79.5)
Diastolic blood pressure * (mmHg), mean (STD)	67.0 (14.5)	75.5 (81.1)
Heart rate * (bpm), mean (STD)	116.5 (20.0)	116.5 (22.6)
Respiratory rate * (bpm), median (IQR)	28 (23–36)	28 (24–34)
Oxygen saturation * (%), median (IQR)	95 (91–97)	95 (90–98)
Temperature (°C), median (IQR)	38.2 (37.1–39.0)	37.8 (36.7–38.7)
HIV-positive, n (%)	117 (80.7)	232 (82.0)
Diagnosis of HIV ^†^, n (%) Previously known New diagnosis Unknown	76 (64.9) 17 (14.5) 24 (20.5)	165 (71.1) 16 (6.9) 51 (22.0)
CD4 ^†*^ (cells/µl), median (IQR)	119 (47-205)	86 (30-185)
Pre-hospital antiretroviral treatment ^‡^, n (%)	60 (79.0)	128 (77.6)
Pre-hospital cotrimoxazole prophylaxis ^‡^, n (%)	59 (77.6)	120 (72.7)
Chest X-ray changes consistent with pneumonia *, n (%)	145 (100)	190 (73.4)
Confirmed diagnosis of tuberculosis *, n (%)	0 (0)	67 (33.2)
In-hospital commencement of tuberculosis treatment *, n (%)	0 (0)	91 (33.0)
Positive malaria rapid diagnostic test *, n (%)	4 (3.0)	5 (1.9)
Positive blood culture *, n (%)	7 (5.3)	20 (7.5)
Positive BinaxNOW *Streptococcus pneumoniae* urinary antigen *, n (%)	34 (25.2)	48 (19.1)

*Missing data was not imputed; ^†^of those who are human immunodeficiency virus-positive; ^‡^of those who were previously known to be HIV-positive. STD: standard deviation; bpm: beats/breaths per minute; IQR: interquartile range.

Overall, 349 provisional cases (81.5%) were HIV-positive, with 33 (9.5%) of those newly diagnosed. One hundred and eighty-eight (78.0%) of those previously known to be HIV-positive were taking antiretroviral treatment prior to admission. One hundred and twenty-four (85.5%) provisional cases reported a previous diagnosis of emphysema and 34 (23.4%) reported previous tuberculosis. Forty-six (10.7%) provisional cases died prior to hospital discharge and a further 18 (5.2%) died following discharge, prior to follow-up. Note that post-discharge mortality was only known for those who had not already been excluded.

### Air pollution monitoring

Ambulatory PM _2.5_ exposure data was available for 379 (95.2%) of participants who completed follow-up, while ambulatory CO, household PM _2.5_, and household CO exposure data were available for 388 (97.5%), 258 (64.8%), and 375 (94.2%) of participants, respectively ( Table 2). Data were missing because of technical faults with the pollution monitoring devices. Median duration between recruitment and monitoring was 11 days (IQR 6–24) and 65 days (IQR 57–83) for ambulatory exposures, and 11 days (IQR 6–23) and 64 days (IQR 57–78) for household exposures, for controls and cases respectively. There was no significant difference in month of exposure monitoring between cases and controls (data not shown).

**Table 2.** Univariate analysis of potential risk factors for pneumonia in HIV-positive and HIV-negative sub-groups.

Exposures	HIV-positive subgroup				HIV-negative subgroup			
	Cases (n= 117)	Controls (n= 169)	Unadjusted OR (95% CI)	p-value	Cases (n= 28)	Controls (n= 84)	Unadjusted OR (95% CI)	p-value
Participant characteristics								
Age (years) ^†^, median (IQR)	36 (31-43)	36 (32-44)	--	--	39 (30-64)	35 (26-42)	--	--
Gender ^†^, n (%) Male (reference) Female	68 (58.1) 49 (41.9)	93 (55.0) 76 (45.0)	-- --	-- --	23 (82.1) 5 (17.9)	54 (64.3) 30 (35.7)	-- --	-- --
Alcohol intake ^††^, n (%) Never (reference) Previous drinker Current drinker	63 (53.9) 46 (39.3) 8 (6.8)	94 (55.6) 40 (23.7) 35 (20.7)	1 **1.72 (1.01-2.92)** **0.34 (0.15-0.78)**	**--** **0.046** **0.011**	11 (39.3) 13 (46.4) 4 (14.3)	52 (61.9) 15 (17.9) 17 (20.2)	1 **4.10 (1.53-11.00)** 1.11 (0.31-3.96)	-- **0.005** 0.869
Smoking status (all forms) ^††^, n (%) Never smoked (reference) Ex-smoker Current smoker	85 (72.7) 28 (23.9) 4 (3.4)	123 (72.8) 27 (16.0) 19 (11.2)	1 1.50 (0.83-2.73) **0.30 (0.10-0.93)**	-- 0.182 **0.036**	13 (26.4) 10 (35.7) 5 (17.9)	68 (81.0) 7 (8.3) 9 (10.7)	1 **7.47 (2.41-12.2)** 2.9 1 (0.84-10.08)	-- **0.001** 0.093
Socioeconomic status quintile ^†††^ Highest (reference) High Middle Low Lowest	19 (16.2) 25 (21.4) 26 (22.2) 22 (18.8) 25 (21.4)	26 (15.4) 40 (23.7) 33 (19.5) 37 (21.9) 33 (19.5)	1 0.86 (0.39-1.86) 1.08 (0.49-2.36) 0.81 (0.37-1.80) 1.04 (0.48-2.28)	-- 0.692 0.851 0.610 0.929	6 (21.4) 2 (7.1) 7 (25.0) 3 (10.7) 10 (35.7)	28 (33.3) 14 (16.7) 15 (17.9) 16 (19.1) 11 (13.1)	1 0.67 (0.12-3.74) 2.18 (0.62-7.66) 0.86 (0.19-3.98) **4.24 (1.24-14.50)**	-- 0.645 0.225 0.863 **0.021**
Participant health characteristics								
Body mass index (kg/m ^2^) ^††^, mean (STD)	19.9 (2.5)	21.6 (3.9)	**0.85 (0.78-0.92)** ^‡^	**< 0.001**	20.9 (3.8)	23.2 (4.9)	**0.84 (0.72-0.98)** ^‡^	**0.023**
CD4 count (cells/µl) ^††^, median (IQR)	129 (49-209)	355 (236-492)	**0.99 (0.99-0.99)** ^‡^	**< 0.001**	--	--		
Antiretroviral therapy ^††^, n (%) No (reference) Yes	49 (41.9) 68 (58.1)	39 (23.1) 130 (76.9)	1 **0.42 (0.25-0.70)**	-- **0.001**	-- --	-- --	-- --	-- --
Cotrimoxazole prophylaxis ^††^, n (%) No (reference) Yes	50 (42.7) 67 (57.3)	42 (24.9) 127 (75.2)	1 **0.44 (0.27-0.73)**	-- **0.002**	-- --	-- --	-- --	-- --
Chronic respiratory disease ^††^, n (%) No (reference) Yes	6 (5.13) 111 (94.9)	89 (52.7) 80 (47.3)	1 **20.58 (8.58-49.38)**	-- **< 0.001**	1 (3.6) 27 (96.4)	67 (79.8) 17 (20.2)	1 **106.41 (13.49-839.66)**	-- **< 0.001**
Previous respiratory diagnosis, n (%) No (reference) Yes	14 (12.0) 103 (88.0)	103 (61.0) 66 (39.1)	1 **11.48 (6.07-21.73)**	-- **< 0.001**	2 (7.1) 26 (92.9)	31 (86.9) 11 (13.1)	1 **86.27 (17.92-415.40)**	-- **< 0.001**
Previous chronic respiratory symptoms, n (%) No (reference) Yes	8 (6.8) 109 (93.2)	92 (54.4) 77 (45.6)	1 **16.28 (7487-35.01)**	-- **< 0.001**	1 (3.6) 27 (96.4)	69 (82.1) 15 (17.9)	1 **124.20 (15.63-986.79)**	-- **< 0.001**
FEV _1_ % of predicted *, median (IQR) (n=349)	60.2 (54.2-73.3)	70.3 (62.0-81.0)	**0.98 (0.96-0.99)** ^‡^	**0.006**	57.0 (41.7-66.0)	71.4 (61.6-79.7)	**0.93 (0.90-0.97)** ^‡^	**< 0.001**
FVC % of predicted *, median (IQR) (n=349)	74.7 (65.4-82.6)	81.5 (71.7-88.6)	**0.97 (0.95-0.99)** ^‡^	**0.001**	73.0 (64.6-80.4)	82.2 (73.1-89.6)	**0.93 (0.89-0.97)** ^‡^	**0.002**
Spirometric classification *, n (%) (n=349) Normal (reference) Obstructive Restrictive	28 (27.5) 17 (16.7) 57 (55.8)	76 (51.7) 17 (11.6) 54 (36.7)	1 **2.71 (1.22-6.04)** **2.87 (1.61 – 5.07)**	-- **0.014** **<0.001**	6 (26.1) 8 (34.8) 9 (39.1)	37 (48.1) 10 (13.0) 30 (39.0)	1 **4.93 (1.39-17.54)** 1.85 (0.59-5.78)	-- **0.014** 0.290
Pollution exposures								
Mean ambulatory PM _2.5_ exposure (µg/m ^3^) ^††^, median (IQR)	60.4 (41.0-103.0)	55.2 (34.6-89.1)	1.00 (1.00-1.00) ^‡^	0.145	70.7 (50.3-109.4)	56.7 (41.3-92.3)	1.00 (1.00-1.01) ^‡^	0.410
Mean Ambulatory CO exposure (ppm) ^††^, median (IQR)	6.0 (2.7-11.3)	4.5 (2.5-9.2)	**1.03 (1.00-1.07)** ^‡^	**0.047**	3.1 (1.2-7.4)	4.7 (1.0-11.5)	0.93 (0.85-1.01) ^‡^	0.079
Mean household PM _2.5_ exposure * (µg/m ^3^) ^†††^, median (IQR) (n=258)	125.2 (77.4-254.9)	167.1 (90.6-311.9)	1.00 (1.00-1.00) ^‡^	0.559	189.5 (132.4-344.3)	132.4 (69.5-292.1)	1.00 (1.00-1.00) ^‡^	0.074
Mean household CO exposure (ppm) ^††^, median (IQR)	6.9 (2.8-13.6)	5.4 (2.9-11.8)	1.02 (1.00-1.04) ^‡^	0.109	4.5 (2.4-8.7)	7.5 (3.6-16.1)	0.96 (0.90-1.01) ^‡^	0.121
Cooking with solid fuel frequency ^††^, n (%) Cooks rarely (reference) Cooks occasionally Cooks sometimes Cooks often Cooks frequently	29 (24.8) 36 (30.8) 40 (34.2) 12 (10.3) 0 (0)	22 (13.0) 53 (31.4) 73 (43.2) 20 (11.8) 1 (0.6)	1 0.52 (0.26-1.03) **0.42 (0.21-0.82)** 0.46 (0.18-1.13) 1	-- 0.062 **0.011** 0.088 --	4 (14.3) 16 (57.1) 8 (28.6) 0 (0) 0 (0)	16 (19.1) 23 (27.4) 34 (40.5) 10 (11.9) 1 (1.2)	1 2.78 (0.78-9.89) 0.94 (0.25-3.59) 1 1	-- 0.114 0.929 -- --
Primary cooking fuel ^†††^, n (%) Electricity (reference Wood Charcoal Plastic Bottles	9 (7.7) 17 (14.5) 91 (77.8) 0 (0)	13 (7.7) 26 (15.4) 130 (76.9) 0 (0)	1 0.94 (0.33-2.69) 1.01 (0.41-2.46) --	-- 0.915 0.981 --	1 (3.6) 8 (28.6) 18 (64.3) 1 (3.6)	10 (11.9) 6 (7.1) 68 (81.0) 0 (0)	1 **13.33 (1.32-134.61)** 2.65 (0.32-22.06) 1	-- **0.028** 0.368 --
Ventilation whilst cooking ^†††^, n (%) Mainly cooks outside or only uses electricity (reference) Mainly cooks inside with ventilation Mainly cooks inside without ventilation	22 (18.8) 73 (62.4) 22 (18.8)	28 (16.5) 108 (63.5) 34 (20.0)	1 0.86 (0.46-1.62) 0.82 (0.38-1.79)	-- 0.641 0.623	2 (7.14) 19 (67.9) 7 (25.0)	14 (16.7) 66 (78.6) 4 (4.8)	1 2.02 (0.42-9.66) **12.25 (1.79-83.95)**	1 0.381 **0.011**
Pollution from heating/lighting ^†††^, n (%) No Yes	105 (89.7) 12 (10.3)	155 (91.2) 15 (8.8)	1 1.18 (0.53-2.62)	-- 0.683	23 (82.1) 5 (17.9)	78 (92.9) 6 (7.1)	1 2.83 (0.79-10.11)	-- 0.110

*Missing data was not imputed. ^‡^ Per unit change. ^†^ *A priori* forced variable included in the logistic regression model. ^††^ *A priori* potential confounder with Likelihood Test Ratio p-value <0.2 therefore entered into the logistic regression model (note PM _2.5_ and CO exposures included as potential confounders for CRD analysis only). ^†††^ *A priori* potential confounder with Likelihood Test Ratio p-value >0.2 therefore not entered into the logistic regression model. OR: odds ratio; CI: confidence interval; IQR: interquartile range; STD: standard deviation; FEV _1_: forced expiratory volume in 1 second; FVC: forced vital capacity; PM _2.5_: particulate matter <2.5µm; CO: carbon monoxide; ppm: parts per million.

### Univariate analysis of potential risk factors

Findings were consistent for pollution assessment modalities in both HIV-positive and HIV-negative subgroups: exposure to ambulatory and household PM _2.5_ and CO had no effect on pneumonia risk with unadjusted ORs of approximately one for all measures of exposure ( Table 2). In the HIV-positive subgroup, there were no consistent findings related to frequency of cooking with solid fuels and no significant findings relating to fuel use, household ventilation, or other forms of pollution exposure ( Table 2 and Supplementary File 2). In the HIV-negative subgroup, cooking with wood (OR 13.33 [95% CI 1.32–134.61, p=0.028]) and cooking inside without ventilation (OR 12.25 [95% CI 1.79–83.95, p=0.011]) were both associated with an increased risk of pneumonia.

CRD was associated with an increased risk of pneumonia in both study groups (HIV-positive: OR 20.58 [95% CI 8.58–49.38], p<0.001; and HIV-negative: OR 106.41 [95% CI 13.49–839.66, p<0.001]). Factors associated with a reduced risk were taking antiretroviral treatment (OR 0.42 [95% CI 0.25–0.70, p=0.001]), increasing BMI (HIV-positive: OR 0.85 [95% CI 0.78–0.92, p<0.001]; HIV-negative: 0.84 [95% CI 0.72–0.98, p=0.023]) and increasing CD4 count (cells/µl) (OR 0.99 [95% CI 0.99–0.99, p<0.001]) ( Table 2). In the HIV-positive group, socioeconomic status was not associated with pneumonia (OR 1.01 [95% CI 0.85–1.20, p=0.943]), but there was an increased risk of pneumonia with decreasing socioeconomic status in the HIV-negative group (OR 1.38 [95% CI 1.02–1.85, p=0.034]). Further potential risk factors and confounding factors are reported in Supplementary File 2.

### Spirometry

Two independent reviewers deemed the spirometry data usable as per American Thoracic Society standards in 349 (87.7%) participants who completed follow-up. The two reviewers agreed on the spirometry interpretation for 99.1% of participants. Of the 91 (72.8%) cases that had abnormal spirometry at their initial follow-up appointment, it was only possible to repeat spirometry in 13 (14.3%) a minimum of 4 months after their pneumonia episode to determine their final spirometry status: spirometry remained abnormal in all these individuals. Pre-bronchodilator percentage of predicted forced expiratory volume in 1 second and forced vital capacity were lower in cases than in controls in both the HIV-positive and HIV-negative subgroups ( Table 2). Restrictive spirometry was a risk factor for pneumonia in the HIV-positive subgroup only (OR 2.87 [95% 1.61–5.07, p<0.001]), whereas obstructive spirometry was predictive of pneumonia in both subgroups (HIV-positive: OR 2.71 [95% CI 1.22–6.04, p=0.014]; and HIV-negative: OR 4.93 [95% CI 1.39-17.54, p=0.014]). Abnormal spirometry was associated with the presence of CRD (composite definition) in the HIV-positive subgroup (Pearson’s chi-square test, p<0.001), but not in the HIV-negative group (p=0.165).

### Multivariate analysis of potential risk factors

After adjustment for confounders, mean ambulatory and household PM _2.5_ and CO exposures were not associated with pneumonia in the HIV-positive or HIV-negative subgroups ( Table 3). CRD had a substantial effect on pneumonia risk in both HIV-positive and HIV-negative subgroups (OR 28.07 [95% CI 9.28–84.83 p<0.001] and OR 104.27 [95% CI 12.86–852.35, p<0.001], respectively). Factors associated with a reduced risk of pneumonia after adjustment for confounders in the HIV-positive subgroup included body mass index (BMI; HIV-positive: OR 0.85 [95% CI 0.75–0.95, p=0.008]), increasing CD4 count (OR 0.99 [95% CI 0.99–0.99, p<0.001]) and antiretroviral therapy (OR 0.23 [95% CI 0.09–0.60, p=0.002]). In the HIV-negative subgroup, after adjustment for age and sex, being an ex-smoker (OR 5.92 [95% CI 1.69–20.79, p=0.006]) and cooking inside without ventilation (OR 9.32 [95% CI 1.24–69.81, p=0.030]) were associated with an increased risk of pneumonia and increasing BMI (OR 0.84 [95% CI 0.72–0.99, p=0.036]) was found to be protective. We did not find evidence of spatial clustering in pneumonia risk, with all p-values for the test on the presence of residual spatial effects being well above 10%.

**Table 3.** Multivariate analysis of the effects of household air pollution exposure and chronic respiratory disease on pneumonia risk in HIV-positive and HIV-negative sub-groups.

Exposures	Adjusted OR (95% CI)	p-value
HIV-positive subgroup		
Mean ambulatory PM _2.5_ exposure (µg/m ^3^) *	1.00 (1.00–1.01)	0.141
Mean ambulatory CO exposure (ppm) *	1.07 (1.00–1.14)	0.052
Mean household PM _2.5_ exposure (µg/m ^3^) ^†§^	1.00 (1.00–1.00)	0.608
Mean household CO exposure (ppm) *	1.03 (1.00–1.07)	0.081
Chronic respiratory disease *	**28.07 (9.29–84.83)**	**< 0.001**
HIV-negative subgroup		
Mean ambulatory PM _2.5_ exposure (µg/m ^3^) ^‡^	1.00 (0.99–1.01)	0.872
Mean ambulatory CO exposure (ppm)‡	0.95 (0.87–1.03)	0.219
Mean household PM _2.5_ exposure (µg/m ^3^) ^‡§^	1.00 (1.00–1.00)	0.307
Mean household CO exposure (ppm) ^‡^	0.96 (0.91–1.02)	0.206
Chronic respiratory disease ^‡^	**104.27 (12.86–852.35)**	**<0.001**

*Adjusted for age, sex, CD4, chronic respiratory disease, antiretroviral treatment, body mass index, occupational status and alcohol intake; ^†^adjusted for age, sex, CD4, chronic respiratory disease and antiretroviral treatment; ^‡^adjusted for age and sex. ^§^Missing household PM _2.5_ data were not imputed; therefore, analyses were restricted to 169 and 79 observations in the HIV–positive and HIV–negative subgroups, respectively. OR: odds ratio; CI: confidence interval; PM _2.5_: particulate matter <2.5µm; CO: carbon monoxide; ppm: parts per million.

## Discussion

We found no association between household air pollution exposure, measured using ambulatory and household monitoring of pollutants, and radiologically confirmed pneumonia in urban HIV-positive Malawian adults. This was consistent when measuring ambulatory and household PM _2.5_ and CO, and self-reported exposures. Similar results were found in an exploratory study of HIV-negative individuals. In contrast, we found a strong association between CRD (defined by participant-reported symptoms and diagnoses), as well as spirometric abnormalities, and pneumonia in both HIV-positive and HIV-negative individuals in this setting.

The AIR study and our earlier BOLD study in Malawi both found a high prevalence of restrictive lung disease ^17^. The underlying etiology, pathology, epidemiology and prognosis of this low FVC phenomenon requires further investigation, particularly since low FVC is associated with increased mortality in other settings ^18, 19^. The relationship identified by AIR between restriction and pneumonia is potentially relevant to our understanding of this increased mortality.

It seems likely that socioeconomic factors explain the unexpected findings of reduced risk of pneumonia in current smokers and consumers of alcohol, as in the context of urban Malawi, the poorest individuals cannot afford cigarettes and alcohol ^20^. The low prevalence of smoking in this setting means that smoking is not a major driver of pneumonia risk in multivariate analysis, unlike in higher resourced countries ^21^.

Reduced BMI was a strong predictor for pneumonia in both subgroups, even after adjustment for CD4 in the HIV-positive subgroup; malnutrition may play a role in pneumonia risk, as has been shown in children ^22^. Further research into nutritional status in this population and possible interventions is warranted.

This is the only study of household air pollution and pneumonia in adults to have used multiple measurements of air pollution exposure with radiologically confirmed hospitalised pneumonia cases. While there is no gold standard method for air pollution monitoring, measuring ambulatory and household levels of two different major components of air pollution (PM _2.5_ and CO) is likely to capture a representative picture of an individual’s total exposure. Mean household PM _2.5_ levels detected (all homes: median 149.5 µg/m ^3^, IQR 85.0–289.0 µg/m ^3^) and mean household CO levels (all homes: median 6.4 ppm, IQR 2.9–12.6ppm) were comparable to those detected in a previous study of urban Malawian homes (mean 150 µg/m ^3^, standard deviation 360 µg/m ^3^ and mean 6.14 ppm, respectively) ^23^. The detected levels of exposure greatly exceed the levels considered safe: WHO Air Quality Guidelines recommend not exceeding 24hour-mean PM _2.5_ levels of 25 µg/m ^3^ ^24^. Mean ambulatory PM _2.5_ levels detected (all participants: median 59.4 µg/m ^3^, IQR 39.6–96.1 µg/m ^3^) equate to a 2.5%–5% increased risk of short-term mortality according to these guidelines.

The latest Global Burden of Disease Study (2013) estimates for the burden of adult ALRI caused by household air pollution are based on data extrapolated from evidence for tobacco smoke and outdoor air pollution ^3^. A systematic review found only a small number of studies, of limited quality, assessing the effects of household air pollution on ALRI in adults, and was thus unable to conclude that an effect exists ^4^. Our findings are inconsistent with evidence presented by Ezzati and Kammen, who demonstrated a dose-dependent relationship between household air pollution exposure and ALRI ^25^. This study from rural Kenya conducted household monitoring prospectively for 12 hours per day over a 2-year period, but did not use radiologically confirmed pneumonia, did not account for HIV status, and their cohort included children over the age of 5 years.

The lack of association between household air pollution and ALRI in adults identified in this study may be explained by the overwhelming effect of other major risk factors (such as CRD, HIV-associated factors and BMI) in this setting. This could explain why an association is evident in children but not adults, in whom lifelong exposure to other factors plays a more important role ^2^. Alternatively, it is possible that we have not detected a true association between household air pollution and ALRI in adults owing to methodological limitations, in particular with exposure assessments.

Although the AIR study is the largest and most detailed study of household air pollution and ALRI in adults to date, it has a number of limitations. We were unable to evaluate HIV-positive and HIV-negative individuals together due to a lack of statistical power, but our findings were broadly consistent across both groups. The original sample size for both groups was not met because recruitment was slower than anticipated leading to lower power to detect an effect. However, in the HIV-positive group, we were able to detect an OR greater than 1.0003 per unit change for ambulatory PM _2.5_ exposure (our primary exposure of interest) with 80% power. Findings in the HIV-negative group are exploratory only. Potential risk factors were assessed after the episode of pneumonia, and so questionnaire assessments may have been subject to recall bias. Our composite assessment of CRD is not validated and may have been vulnerable to recall bias, although our findings are corroborated by spirometric data. Objective measurements of air pollution exposures were made, but these may not be representative of pre-pneumonia exposures, although 138/142 (97.2%) cases reported that they had returned to normal levels of function. A sensitivity analysis, in which cases without reported full functional recovery were excluded, also found no effects of mean ambulatory PM _2.5_ exposure (data not shown). In addition, our exposure monitoring does not account for differences in exposure over the life course. The ambulatory pollutant monitoring is unable to distinguish between outdoor and indoor exposures, although since our findings are consistent across ambulatory, household, and questionnaire assessments, we argue that our findings are reflective of the effects of household air pollution.

Pneumonia is a major health burden in sub-Saharan Africa ^3^. Although there are compelling reasons for tackling household air pollution ^26^, other issues need to be addressed to reduce the burden of pneumonia in adults. Evidence from this study can be used to establish global estimates for the contribution of household air pollution exposure to the burden of disease, to ensure the limited available resources for public health interventions are appropriately directed. Risk factors associated with pneumonia in this study, such as HIV and BMI, are typically associated with socioeconomic status, indicating that poverty is an important driver of pneumonia in urban African adults. To reduce the burden of pneumonia, further research into the effects of CRD and the underlying etiologies in this setting are required. Prenatal and childhood malnutrition may play a role. Targeted evidence-based strategies to reduce the high burden of CRD seen in young adults are needed and may help to tackle the high morbidity and mortality caused by pneumonia.

## Data availability

The raw dataset for the AIR study is available on OSF: http://doi.org/10.17605/OSF.IO/G95KQ ^27^. This dataset does not include data for seven participants (two of who completed the full study), as they did not give permission for their data to be shared publically. Applications by *bona fide* researchers can be made to the relevant Research and Ethics Committees (College of Medicine Research Ethics Committee, University of Malawi, and the Liverpool School of Tropical Medicine Research Ethics Committee [ lstmrec@lstmed.ac.uk]) to access the full dataset. Requests will be facilitated through the corresponding author ( hannah.jary@lstmed.ac.uk).

## Supplementary material

Supplementary File 1: AIR Study Questionnaires for Follow-up Appointments (AIR Follow-up questionnaire and Edited BOLD Questionnaire).

Supplementary File 2: Additional univariate analysis of exposures for HIV-positive and HIV-negative subgroups.
