# Supplementary material for: Household air pollution, chronic respiratory disease and pneumonia in Malawian adults: A case-control study
Source: Wellcome Open Res. 2017 Oct 24;2:103. [Version 1] doi: 10.12688/wellcomeopenres.12621.1 (PMC5730861; doi:10.12688/wellcomeopenres.12621.1)
Supplement: Supplementary file 1 [file wellcomeopenres-2-13666-s0000.tgz › 3e4073dd-02c8-4c33-acd4-365618092393.docx]

**AIR Study: Follow Up Questionnaire**

AIR Number: Initials: Barcode:

| 1. **CONTINUED ELIGIBILITY CHECK LIST** | | | |
| --- | --- | --- | --- |
| 1.1 | Is the participant still alive ?  *If the answer is ‘No’ then do not proceed. Complete Section 7.* | **Y** | **N** |
| 1.2 | Has the participant been started on TB medications since recruitment?  *If the answer is ‘Yes’ then do not proceed. Complete Section 7.* | **Y** | **N** |
| 1.2 | Is the participant currently free from pneumonia-like symptoms?  *If the answer is ‘No’ then do not proceed. Complete Section 7.* | **Y** | **N** |
| 1.1 | Does the participant have a valid completed consent form and give verbal consent to remain in the study?  *If the answer is ‘No’ then do not proceed. Complete Section 7.* | **Y** | **N** |

***Cases: Please think back to how your life was 6 months ago (prior to becoming unwell with pneumonia). When answering the questions below, try to answer for how life was around that time.***

***Controls: Please think back to how your life was 6 months ago. When answering the questions below, try to answer for how life was around that time.***

| 1. **PERSONAL AND HOUSEHOLD DETAILS** | | | | | | | | | | | | | | | | | | | | | | | | | | | | | | | | | | | | | | | | | | | | | | | | | | | | | |
| --- | --- | --- | --- | --- | --- | --- | --- | --- | --- | --- | --- | --- | --- | --- | --- | --- | --- | --- | --- | --- | --- | --- | --- | --- | --- | --- | --- | --- | --- | --- | --- | --- | --- | --- | --- | --- | --- | --- | --- | --- | --- | --- | --- | --- | --- | --- | --- | --- | --- | --- | --- | --- | --- |
| 1.1 | Marital status: | | | | | | | | | | | | | | | | | | | | | | | | | | | | | | | | | | | | | | | | | | | | | | | | | | | | |
|  | **Single** | | |  | | | | **Married** | | | | | | |  | | | **Separated** | | | | | | |  | | | | | **Divorced** | | | | | | |  | | | | | **Widowed** | | | | | | | | | |  | |
| 1.2 | Are you currently? | | | | | | | | | | | | | | | | | | | | | | | | | | | | | | | | | | | | | | | | | | | | | | | | | | | | |
|  | **Paid employee** | | | | | | | | | | | | | | | | | |  | | **Unpaid family worker** | | | | | | | | | | | | | | | | | | | | | | | | | | | | | |  | | |
|  | **Paid domestic worker** | | | | | | | | | | | | | | | | | |  | | **Student** | | | | | | | | | | | | | | | | | | | | | | | | | | | | | |  | | |
|  | **Self-employed** | | | | | | | | | | | | | | | | | |  | | **Other** | | | | | | | | | | | | | | | | | | | | | | | | | | | | | |  | | |
|  | **Unemployed** | | | | | | | | | | | | | | | | | |  | |  | | | | | | | | | | | | | | | | | | | | | | | | | | | | | | | | |
| 1.3 | What is your current occupation? | | | | | | | | | | | | | | | | | | | | | | | | | | | | | | | | | | | | | | | | | |  | | | | | | | | | | |
| 1.4 | How many children in your household are eligible for school? | | | | | | | | | | | | | | | | | | | | | | | | | | | | | | | | | | | | | | | | | | | | | | | | | | |  | |
| 1.5 | How many children in your household are going to school? | | | | | | | | | | | | | | | | | | | | | | | | | | | | | | | | | | | | | | | | | | | | | | | | | | |  | |
| 1.6 | How many children in your household are age 5 or younger? | | | | | | | | | | | | | | | | | | | | | | | | | | | | | | | | | | | | | | | | | | | | | | | | | | |  | |
| 1.7 | Which of these best describes the material the roof of your house is made from? | | | | | | | | | | | | | | | | | | | | | | | | | | | | | | | | | | | | | | | | | | | | | | | | | | | | |
|  | **Natural Roof** | | | | No roof | | | | |  | Thatch / Palm Leaf | | | | | | | |  | Sod | | | | | | | |  | | |  | | | | | | | | |  |  | | | | | | | |  | | |  | |
|  | **Rudimentary Roof** | | | | Rustic Mat | | | | |  | Palm / Bamboo | | | | | | | |  | Wooden Planks | | | | | | | |  | | | Cardboard | | | | | | | | |  |  | | | | | | | |  | | |  | |
|  | **Finished Roof** | | | | Metal | | | | |  | Wood | | | | | | | |  | Calamine / Cement Fibre / Ceramic tiles / Cement / Roofing shingles | | | | | | | |  | | |  | | | | | | | | |  | Other (specify) | | | | | | | |  | | |  | |
| 1.8 | Which of these best describes the material the walls of your house are made from? | | | | | | | | | | | | | | | | | | | | | | | | | | | | | | | | | | | | | | | | | | | | | | | | | | | | |
|  | **Natural Walls** | | | | | No walls | | |  | Cane / Palm / Trunks | | | | | | |  | | Dirt | | | | |  | | | | |  | | | | |  | | | | |  | | | | | | | |  | |  | | | |  |
|  | **Rudimentary Walls** | | | | | Bamboo with mud | | |  | Stone with mud | | | | | | |  | | Uncovered mud brick | | | | |  | | | | | Plywood | | | | |  | | | | | Cardboard | | | | | | | |  | | Reused wood | | | |  |
|  | **Finished Walls** | | | | | Cement / Cement bricks | | |  | Stone with Lime / Cement | | | | | | |  | | Bricks | | | | |  | | | | | Covered mud bricks | | | | |  | | | | | Wood planks / shingles | | | | | | | |  | | Other (specify) | | | |  |
| 1.9 | Which of these best describes the material the floor inside your house is made from? | | | | | | | | | | | | | | | | | | | | | | | | | | | | | | | | | | | | | | | | | | | | | | | | | | | | |
|  | **Natural Floor** | | Earth / Sand | | | | | |  | Dung | | | | | | | | |  |  | | | | | |  |  | | | | | |  | | |  | | | | | | | |  | |  | | | | | | | |
|  | **Rudimentary Floor** | | Wood Planks | | | | | |  | Palm / Bamboo | | | | | | | | |  |  | | | | | |  |  | | | | | |  | | |  | | | | | | | |  | |  | | | | | | | |
|  | **Finished Floor** | | Parquet or polished wood | | | | | |  | Vinyl or ashphalt strips | | | | | | | | |  | Ceramic Tiles | | | | | |  | Cement | | | | | |  | | | Carpet | | | | | | | |  | | Other (specify) | | | | | | | |
| 1.10 | Which best describes the windows in your home? | | | | | | | | | | | | | | | | | | | | | | | | | | | | | | | | | | | | | | | | | | | | | | | | | | | | |
|  | **No windows** |  | | | | | **Glass windows** | | | | |  | | | | **Space only** | | | | |  | **Space covered with grass** | | | | | | | | | |  | | | **Other**  **(specify)** | | | | | | | | | | | | | | | | | | |
| 1.11 | Does your household own any livestock, herds, other farm animals or poultry ? | | | | | | | | | | | | | | | | | | | | | | | | | | | | | | | | | | | | | | **Don’t know** | | | | | | **Y** | | | | | **N** | | | |
| 1.12 | How many of the following animals does this household own? | | | | | | | | | | | | | | | | | | | | | | | | | | | | | | | | | | | | | | | | | | | | | | | | | | | | |
|  | **Cattle** | | | | | | | | | | | |  | | | | | | | | | **Horses, donkeys or mules** | | | | | | | | | | | | | | | | | | | | | | | | | | | |  | | | |
|  | **Milk cows or bulls** | | | | | | | | | | | |  | | | | | | | | | **Sheep** | | | | | | | | | | | | | | | | | | | | | | | | | | | |  | | | |
|  | **Chickens** | | | | | | | | | | | |  | | | | | | | | | **Dogs** | | | | | | | | | | | | | | | | | | | | | | | | | | | |  | | | |
|  | **Pigs** | | | | | | | | | | | |  | | | | | | | | | **Cats** | | | | | | | | | | | | | | | | | | | | | | | | | | | |  | | | |
|  | **Goats** | | | | | | | | | | | |  | | | | | | | | | **Other (specify)** | | | | | | | | | | | | | | | | | | | | | | | | | | | |  | | | |
| 1.13 | Do any of these animals sleep in the same room as you? | | | | | | | | | | | | | | | | | | | | | | | | | | | | | | | | | | | | | | | | | | | | | | | | **Y** | | | **N** | |
| 1.14 | Does any member of your household have a bank account? | | | | | | | | | | | | | | | | | | | | | **Declined to answer** | | | | | | | | | | | | | | | | | | | **Don’t know** | | | | | | | | **Y** | | | **N** | |
| 1.15 | What toilet facilities are there ? | | | | | | | | | | | | | | | | | | | | | | | | | | | | | | | | | | | | | | | | | | | | | | | | | | | | |
|  | **None** | | | | | | | | | | | | **Simple Pit Latrine** | | | | | | | | | **Ventilated Improved Pit (VIP)** | | | | | | | | | | | | | | | | | | | | | | | | | | | | **Water toilet** | | | |
| 1.16 | What is the source of water for drinking? | | | | | | | | | | | | | | | | | | | | | | | | | | | | | | | | | | | | | | | | | | | | | | | | | | | | |
|  | **Tap to house** | | | | | | | | | | | | **Shared communal tap** | | | | | | | | | **Bore hole** | | | | | | | | | | | | | | | | | | | | | | | | | | | | **Covered well** | | | |
|  | **Open well** | | | | | | | | | | | | | **Lake / River** | | | | | | | | | **Other (specify)** | | | | | | | | | | | | | | | | | | | | | | | | | | |  | | | |
| 1.17 | Since this time last year have there been times when the household did not have enough money to buy bathing soap? | | | | | | | | | | | | | | | | | | | | | | | | | | | | | | | | | | | | | **Declined to answer** | | | | | | | | | | **Y** | | | | | |
|  |  |  |  |  |  |  |  |  |  |  |  |  |  |  |  |  |  |  |  |  |  |  |  |  |  |  |  |  |  |  |  |  |  |  |  |  |  | **Don’t know.** | | | | | | | | | | **N** | | | | | |
| 1.18 | How many mosquito nets does you household have? | | | | | | | | | | | | | | | | | | | | | | | | | | | | | | | | | | | | |  | | | | | | | | | | | | | | | |

***Please think back to how your life was 6 months ago. When answering the questions below, try to answer for how life was around that time.***

| 1. **PERSONAL EXPOSURES** | | | | | | | | | | | | | | | | | | | | | | | | |
| --- | --- | --- | --- | --- | --- | --- | --- | --- | --- | --- | --- | --- | --- | --- | --- | --- | --- | --- | --- | --- | --- | --- | --- | --- |
| 2.1 | Have you ever had a job in a smoky / dusty environment? | | | | | | | | | | | | | | | | **Don’t know** | | | | **Y** | | | **N** |
| 2.2 | If yes, what was that job? | | | | | | | | | | | | | | | |  | | | | | | | |
| 2.3 | Do you still do that job? | | | | | | | | | | | | | | | | | | | | **Y** | | | **N** |
| 2.4 | How many years in total did you do that job for? | | | | | | | | | | | | | | | | | | | |  | | | |
| 2.5 | Do you care for anybody who has a chronic illness? | | | | | | | | | | | | | | | | **Don’t know** | | | | **Y** | | | **N** |
| 2.6 | Have you ever drunk alcohol?  *If no, skip to 2.12* | | | | | | | | | | | | | | | | | | | | **Y** | | | **N** |
| 2.7 | How old where you when you first started drinking alcohol? | | | | | | | | | | | | | | | | | | | |  | | | |
| 2.8 | Do you still drink alcohol?  *If yes, skip to 2.10* | | | | | | | | | | | | | | | | | | | | **Y** | | | **N** |
| 2.9 | How old where you when you stopped drinking alcohol? | | | | | | | | | | | | | | | | | | | |  | | | |
| 2.10 | When drinking, what do you / did you tend to drink? *(tick all that apply)* | | | | | | | | | | | | | | | | | | | | | | | |
|  | **Beer** |  | | **Local Spirits** | | |  | **Spirits** | | | |  | | **Wine** | |  | | **Other (specify)** | | | |  | | |
| 2.11 | When you drank at your heaviest, how often did you tend to drink? *(tick one)* | | | | | | | | | | | | | | | | | | | | | | | |
|  | **Less than once a month** |  | | **1-3 times per month** | | |  | **1-3 times per week** | | | |  | | **4-6 times per week** | |  | | **Everyday** | | | | | |  |
| 2.12 | How often do you usually cook for yourself or your household? *(tick one)* | | | | | | | | | | | | | | | | | | | | | | | |
|  | **Less than once a month** |  | | | **1-3 times per month** | |  | | **1-3 times per week** | | |  | **4-6 times per week** | | |  | | | **Everyday** | | | | |  |
| 2.13 | On days when you cook, how many hours per day do you typically spend cooking? *(tick one)* | | | | | | | | | | | | | | | | | | | | | | | |
|  | **Less than 1 hour** | |  | | | **Between 1 -3 hours** | | | |  | **Between 3 – 6 hours** | | | |  | **More than 6 hours** | | | | | | | |  |
| 2.14 | What type of fire/stove is used in your household for cooking? *(tick all that apply but star the one that is used most often)* | | | | | | | | | | | | | | | | | | | | | | | |
|  | **Open 3-stone fire** | | | | | | | |  | | **Gas cooker** | | | | | | | | | | | |  | |
|  | **Permanent clay cookstove** | | | | | | | |  | | **Electric cooker** | | | | | | | | | | | |  | |
|  | **Portable clay cookstove** | | | | | | | |  | | **Other (specify)** | | | | | | | | |  | | | | |
|  | **Metal cookstove** | | | | | | | |  | | **Don’t know** | | | | | | | | | | | |  | |
| 2.15 | What type of fuel is used in your household for cooking? *(tick all that apply but star the one that is used most often)* | | | | | | | | | | | | | | | | | | | | | | | |
|  | **Wood** | | | | | | | |  | | **Kerosene** | | | | | | | | | | | |  | |
|  | **Charcoal** | | | | | | | |  | | **Gas** | | | | | | | | | | | |  | |
|  | **Plant matter / Crop residue** | | | | | | | |  | | **Electricity** | | | | | | | | | | | |  | |
|  | **Animal dung** | | | | | | | |  | | **Other (specify)** | | | | | | | | |  | | | | |
|  | **Coal** | | | | | | | |  | | **Don’t know** | | | | | | | | | | | |  | |
|  | **Utuchi** | | | | | | | |  | |  | | | | | | | | | | | |  | |
| 2.16 | Where does the cooking for your household take place during the dry season? *(tick all that apply but star the one that is used most often)* | | | | | | | | | | | | | | | | | | | | | | | |
|  | **Inside the living area inside your main house** | | | | | | | |  | | **In a separate kitchen building (separate from the main house)** | | | | | | | | | | | |  | |
|  | **Inside a separate kitchen inside your main house** | | | | | | | |  | | **Other (specify)** | | | | | | | | |  | | | | |
|  | **Outside** | | | | | | | |  | | **Don’t know** | | | | | | | | | | | |  | |
| 2.17 | Where does the cooking for your household take place during the wet season? *(tick all that apply but star the one that is used most often)* | | | | | | | | | | | | | | | | | | | | | | | |
|  | **Inside the living area inside your main house** | | | | | | | |  | | **In a separate kitchen building (separate from the main house)** | | | | | | | | | | | |  | |
|  | **Inside a separate kitchen inside your main house** | | | | | | | |  | | **Other (specify)** | | | | | | | | |  | | | | |
|  | **Outside** | | | | | | | |  | | **Don’t know** | | | | | | | | | | | |  | |
| 2.18 | If you cook indoors, does the room you cook in have any of the following? *(tick all that apply)* | | | | | | | | | | | | | | | | | | | | | | | |
|  | **Windows that open** | | | | | | | |  | | **Other form of ventilation (specify)** | | | | | | | | |  | | | | |
|  | **Chimney** | | | | | | | |  | | **Don’t know** | | | | | | | | | | | |  | |
|  | **Extractor fan** | | | | | | | |  | | **Not applicable (cooks outside)** | | | | | | | | | | | |  | |
| 2.19 | What fuel is used in your household for heating? *(tick all that apply but star the one that is used most often)* | | | | | | | | | | | | | | | | | | | | | | | |
|  | **Wood** | | | | | | | |  | | **Utuchi** | | | | | | | | | | | |  | |
|  | **Charcoal** | | | | | | | |  | | **Gas** | | | | | | | | | | | |  | |
|  | **Plant matter / Crop residue** | | | | | | | |  | | **Electricity** | | | | | | | | | | | |  | |
|  | **Animal dung** | | | | | | | |  | | **Other (specify)** | | | | | | | | |  | | | | |
|  | **Coal** | | | | | | | |  | | **None – never use heating** | | | | | | | | | | | |  | |
|  | **Kerosene** | | | | | | | |  | | **Don’t know** | | | | | | | | | | | |  | |
| 2.20 | What type of lighting is used your household? *(tick all that apply but star the one that is used most often)* | | | | | | | | | | | | | | | | | | | | | | | |
|  | **Battery operated torch** | | | | | | | |  | | **Electricity** | | | | | | | | | | | |  | |
|  | **Simple Paraffin lantern** | | | | | | | |  | | **Other (specify)** | | | | | | | | |  | | | | |
|  | **Hurricane lamp** | | | | | | | |  | | **None – never use lighting** | | | | | | | | | | | |  | |
|  | **Candles** | | | | | | | |  | | **Don’t know** | | | | | | | | | | | |  | |
| 2.21 | In your lifetime (including childhood), which of these fuels have been used regularly in your household for more than 6 months of your life? *(tick all that apply)* | | | | | | | | | | | | | | | | | | | | | | | |
|  | **Wood** | | | | | | | |  | | **Kerosene** | | | | | | | | | | | |  | |
|  | **Charcoal** | | | | | | | |  | | **Gas** | | | | | | | | | | | |  | |
|  | **Plant matter / Crop residue** | | | | | | | |  | | **Electricity** | | | | | | | | | | | |  | |
|  | **Animal dung** | | | | | | | |  | | **Coal** | | | | | | | | | | | |  | |
|  | **Wood shavings** | | | | | | | |  | |  | | | | | | | | | | | |  | |

***Complete the edited BOLD questionnaire before proceeding. When completing the BOLD questionnaire, please think back to how your life was 6 months ago and try to answer for how life was around that time.***

| 1. **HIV HISTORY** | | | | | | |
| --- | --- | --- | --- | --- | --- | --- |
| 3.1 | Have you ever had a HIV test (prior to this study)?  *If no or don’t know, skip to Section 4.* | | | **Don’t know** | **Y** | **N** |
| 3.2 | Have you ever previously tested positive?  *If no, skip to Section 4.* | | | | **Y** | **N** |
| 3.3 | When did you first test positive? | | |  | | |
| 3.4 | Have you started ART?  *If no or don’t know, skip to 3.7* | | | **Don’t know** | **Y** | **N** |
| 3.5 | Which regimen are you taking? | | | | | |
|  | **1A (T30)** |  | **6A** | | |  |
|  | **2A** |  | **7A** | | |  |
|  | **3A** |  | **8A** | | |  |
|  | **4A** |  | **Don’t know** | | |  |
|  | **5A** |  |  | | |  |
| 3.6 | How long have you been on ART for? | | | | | |
|  | **<3 months** | | | | |  |
|  | **3-12 months** | | | | |  |
|  | **>12 months** | | | | |  |
| 3.7 | Are you currently taking co-trimoxazole prophylaxis? | | | **Don’t know** | **Y** | **N** |

***Please think back to how your life was 6 months ago. When answering the questions below, try to answer for how life was around that time.***

| 1. **OTHER MEDICAL HISTORY** | | | | |
| --- | --- | --- | --- | --- |
| 4.1 | As a child, were you ever admitted to hospital for malnutrition? | **Don’t know** | **Y** | **N** |
| 4.2 | As a child, were you admitted to hospital on more than one occasion for breathing problems? | **Don’t know** | **Y** | **N** |
| 4.3 | As a child, were you ever diagnosed with pneumonia? | **Don’t know** | **Y** | **N** |
| 4.4 | As an adult, have you ever been diagnosed with pneumonia? | **Don’t know** | **Y** | **N** |
| 4.5 | Has a doctor or a nurse ever told you that you have : | | | |
|  | Cancer | **Don’t know** | **Y** | **N** |
|  | Chronic kidney disease | **Don’t know** | **Y** | **N** |
|  | Chronic liver disease | **Don’t know** | **Y** | **N** |
|  | Epilepsy | **Don’t know** | **Y** | **N** |
|  | Dementia | **Don’t know** | **Y** | **N** |
| 4.6 | Have you previously received: | | | |
|  | Pneumococcal vaccination | **Don’t know** | **Y** | **N** |
|  | *Haemophilus influenzae* B vaccination | **Don’t know** | **Y** | **N** |
|  | Influenza vaccination within the last year | **Don’t know** | **Y** | **N** |
| 4.7 | Are you pregnant? | **N / A (male)** | **Y** | **N** |

***Please think back to how your life was 6 months ago. When answering the questions below, try to answer for how life was around that time.***

| 1. **MEDICATIONS** | | | | | |
| --- | --- | --- | --- | --- | --- |
| 5.1 | Other than medications for your breathing that we have already asked about or any HIV medications (ART), do you take any other medications?  *If no or don’t know, skip to Section 6.* | | **Don’t know** | **Y** | **N** |
| 5.2 | Give details of these medications. Please tell us about all tablets, liquids, powders, inhalers, creams, injections etc that you are supposed to take. | | | | |
|  | Name of medication | What condition do you take this for? (If name of condition not known, write down main symptoms) | | | |
|  |  |  | | | |
|  |  |  | | | |
|  |  |  | | | |
|  |  |  | | | |
|  |  |  | | | |
|  |  |  | | | |
|  |  |  | | | |
|  |  |  | | | |
|  |  |  | | | |
|  |  |  | | | |

| 1. **FUNCTION (CASES ONLY)** | | | |
| --- | --- | --- | --- |
| 6.1 | Do you feel you have returned to your normal level of function since your episode of pneumonia? (ie. do you feel you are able to do as much now as you could before you had pneumonia?) | **Y** | **N** |
| 6.2 | Have you returned to your usual work / study / daily activities since your episode of pneumonia? | **Y** | **N** |
| 6.3 | Are your cooking habits the same now as they were before your episode of pneumonia?  *If yes, skip to Section 7* | **Y** | **N** |
| 6.4 | Do you cook more or less now that you did before your episode of pneumonia? | **More** | **Less** |

| 1. **FORM COMPLETION** | | |
| --- | --- | --- |
| 7.1 | Date Form Completed |  |
| 7.2 | Initials of AIR Study Team Member |  |

**AIR Study: Edited BOLD Questionnaire**

AIR Number: Initials: Barcode:

##### *Demographics*

1. How many years of schooling have you completed? ___ ___

2a. What is the highest level of schooling you have Primary School 

completed? Middle School 

High School 

Some College (Trade/Professional/Community) 

Four-Year College/University 

None 

Unknown 

2b. What is the highest level of schooling your father Primary School 

has completed? Middle School 

High School 

Some College (Trade/Professional/Community) 

Four Year College/University 

None 

Unknown 

2c. What is the highest level of schooling your mother Primary School 

has completed? Middle School 

High School 

Some College (Trade/Professional/Community) 

Four Year College/University 

None 

Unknown 

3. Please tell me whether this household or any person who lives in the household

has/owns the following items:

READ EACH ITEM: YES NO DON’T KNOW

a. Electricity? ..........................................

b. Flush toilet? ...........................................

c. Fixed telephone? ...................................

d. Cell telephone? ....................................

e. Television? ............................................

f. Radio? ................................................

g. Refrigerator? .........................................

h. Car? ......................................................

i. Moped/scooter/motorcycle? ..................

j. Washing machine? ................................

k. Own their own home?..............................

l. Indoor bath or shower?............................

m. Indoor tap?..............................................

n. Outdoor tap of their own?.........................

o. Bed with mattress?…………………………………

p. Mosquito net?………………………………………..

q. In the last year did you or any person who lives in the household ever go hungry for lack of money? 

most days

most weeks

most months

certain times of the year

occasionally

 never................................

4. When you were 5 years old did any person who lived in your household have/own the following items:

READ EACH ITEM: YES NO DON’T KNOW

a. Electricity? ..........................................

b. Flush toilet? ...........................................

c. Fixed telephone? ...................................

d. Cell telephone? ....................................

e. Television? ............................................

f. Radio? ................................................

g. Refrigerator? .........................................

h. Car? ......................................................

i. Moped/scooter/motorcycle? ..................

j. Washing machine? ................................

k. Own their own home?..............................

l. Indoor bath or shower?............................

m. Indoor tap?..............................................

n. Outdoor tap of their own?.........................

o. Bed with mattress?…………………………………

p. Mosquito net?………………………………………..

q. Ever go hungry for lack of money? 

most days

most weeks

most months

certain times of the year

occasionally

 never................................

5. How many people live in your house with you (including you) __ __

6. How many rooms are there in your house? (excluding kitchen and bathroom/s) __ __

## Respiratory Symptoms and Disorders

These questions pertain mainly to your chest. Please answer yes or no if possible. If you are in doubt about whether your answer is yes or no, please answer no.

### Cough

7. Do you usually cough when you don’t have a cold? Yes 

No 

*[If* ***yes****, continue with Question 7A; If* ***no****, skip to Question 8]*

7A. Are there months in which you cough on most days? Yes 

No 

*[If* ***yes****, ask both Questions 7B & 7C; If* ***no****, skip to Question 8]*

7B. Do you cough on most days for as much as three Yes 

months each year? No 

7C. For how many years have you had this cough? Less than 2 years 

2-5 years 

More than 5 years 

### Phlegm

8. Do you usually bring up phlegm from your chest, or do you usually Yes 

have phlegm in your chest that is difficult to bring up when you No 

don’t have a cold?

*[If* ***yes****, continue with Question 8A; If* ***no****, skip to Question 9]*

8A. Are there months in which you have this phlegm on most Yes 

days? No 

*[If* ***yes****, ask both Questions 8B & 8C; If* ***no****, skip to Question 9]*

8B. Do you bring up this phlegm on most days for as much Yes 

as three months each year? No 

8C. For how many years have you had this phlegm? Less than 2 years 

2-5 years 

More than 5 years 

### Wheezing/Whistling

9. Have you had wheezing or whistling in your chest at any Yes 

time in the last 12 months? No 

*[If* ***yes****, ask both Questions 9A & 9B; If* ***no****, skip to Question 10]*

9A. In the last 12 months, have you had this wheezing Yes 

or whistling only when you have a cold? No 

9B. In the last 12 months, have you ever had an attack of wheezing Yes  or whistling that has made you feel short of breath? No 

### Breathlessness

10. Are you unable to walk due to a condition other than shortness Yes 

of breath? No 

[*If* ***yes*** *to Question 10, please describe this condition on the line below and then skip to*

*Question 12. If* ***no*** or unsure*, go directly to Question 11.]*

Nature of condition(s):

11. Are you troubled by shortness of breath when hurrying on the Yes 

level or walking up a slight hill? No 

*[If* ***yes****, ask Question 11A through 11D; If* ***no****, skip to Question 12]*

11A. Do you have to walk slower than people of your age on Yes 

level ground because of shortness of breath? No 

Does not apply 

11B. Do you ever have to stop for breath when walking at Yes 

your own pace on level ground? No 

Does not apply 

11C. Do you ever have to stop for breath after walking Yes 

about 100 yards (or after a few minutes) on level No 

ground? Does not apply 

11D. Are you too short of breath to leave the house or Yes 

short of breath on dressing or undressing? No 

Does not apply 

12. Has a doctor or other health care provider ever told Yes 

you that you have emphysema? No 

13. Has a doctor or other health care provider ever told you that Yes 

you have asthma, asthmatic bronchitis or allergic bronchitis? No 

*[If* ***yes****, ask Question 13A. If* ***no****, skip to Question 14]*

13A. Do you still have asthma, asthmatic bronchitis or Yes 

allergic bronchitis? No 

14. Has a doctor or other health care provider ever told you that Yes 

you have chronic bronchitis? No 

*[If* ***yes****, ask Question 14A. If* ***no****, skip to Question 15]*

14A. Do you still have chronic bronchitis? Yes 

No 

15. Has a doctor or other health care provider ever told you that Yes 

you have chronic obstructive pulmonary disease (COPD)? No 

***Management Section***

Now I am going to ask you about medicines that you may be taking to help with your breathing. I want to know about medicines that you take on a

regular basis and medicines that you may take only for the relief of symptoms. I would like you to tell me each medicine that you take, what form

do you take it in, and how often you take it each month.

16. In the past 12 months, have you taken any medications for your breathing (including medications for nasal congestion)? Yes 

No 

| 16A.MedicationName (not entered) |  |  |  |  |  |  |  |
| --- | --- | --- | --- | --- | --- | --- | --- |
| 16B.MedicationCode | ___ ___ ___ | **___ ___ _**__ | **___ ___ _**__ | **___ ___ _**__ | **___ ___ _**__ | **___ ___ _**__ | **___ ___ _**__ |
| 16C.Formulation | Pills  ❑  ❑  ❑  ❑  ❑  ❑  ❑  Inhaler  Nebulizer  Liquid  Suppository  Injection  Other | Pills  ❑  ❑  ❑  ❑  ❑  ❑  ❑  Inhaler  Nebulizer  Liquid  Suppository  Injection  Other | Pills  ❑  ❑  ❑  ❑  ❑  ❑  ❑  Inhaler  Nebulizer  Liquid  Suppository  Injection  Other | Pills  ❑  ❑  ❑  ❑  ❑  ❑  ❑  Inhaler  Nebulizer  Liquid  Suppository  Injection  Other | Pills  ❑  ❑  ❑  ❑  ❑  ❑  ❑  Inhaler  Nebulizer  Liquid  Suppository  Injection  Other | Pills  ❑  ❑  ❑  ❑  ❑  ❑  ❑  Inhaler  Nebulizer  Liquid  Suppository  Injection  Other | Pills  ❑  ❑  ❑  ❑  ❑  ❑  ❑  Inhaler  Nebulizer  Liquid  Suppository  Injection  Other |
| **16D.Is the Medicine taken**  **on most days, or just when**  **you have symptoms, or**  **both?** *(If ‘most days’ ask Q16E, if ‘symptoms’ ask Q16F, if ‘both’,*  *ask both Q16E and Q16F.)* | ❑  ❑  ❑  ❑  Most Days  Symptoms  Both  Other | ❑  ❑  ❑  ❑  Most Days  Symptoms  Both  Other | ❑  ❑  ❑  ❑  Most Days  Symptoms  Both  Other | ❑  ❑  ❑  ❑  Most Days  Symptoms  Both  Other | ❑  ❑  ❑  ❑  Most Days  Symptoms  Both  Other | ❑  ❑  ❑  ❑  Most Days  Symptoms  Both  Other | ❑  ❑  ❑  ❑  Most Days  Symptoms  Both  Other |
| 16E. When you are taking the medication, how many days a week do you take it? | __days/week | ______days/week | ______days/week | ______days/week | ______days/week | ______days/week | ______days/week |
| 16F. When you are taking the medication, how many months in the past 12 months have you taken it? | 0-3   4-6   7-9   10-12  | 0-3   4-6   7-9   10-12  | 0-3   4-6   7-9   10-12  | 0-3   4-6   7-9   10-12  | 0-3   4-6   7-9   10-12  | 0-3   4-6   7-9   10-12  | 0-3   4-6   7-9   10-12  |

*If participant does not take any medications to help their breathing, skip to Question 17.*

17. Please tell me about any other products that you take or things you do to help your breathing that you have not already told me about.

| Medicine or Activity | Code |
| --- | --- |
|  | ____ ____ ____ |
|  | ____ ____ ____ |
|  | ____ ____ ____ |
|  | ____ ____ ____ |

19. Have you ever had a period when you had breathing problems Yes 

that got so bad that they interfered with your usual daily No 

activities or caused you to miss work?

*[If* ***yes****, ask Question 19A. If* ***no****, skip to Question 20]*

19A. How many such episodes have you had in the past ____ ____ ____ episodes

12 months?

*[If 19A >0, ask Questions 19B and 19C, else skip to Question 20]*

19B. For how many of these episodes did you need to _____ _____ episodes

see a doctor or other health care provider in the

past 12 months?

19C. For how many of these episodes were you hospitalized _____ _____ episodes

overnight in the past 12 months?

*[If 19C >0, ask Question 19C1, else skip to Question 20]*

19C1. All together, for how many total days were you ____ ____ ____ days

hospitalized overnight for breathing problems in

the past 12 months?

***Smoking***

20. Now I am going to ask you about smoking. First I will ask about cigarettes, including hand rolled cigarettes, and then I will ask about other items that are smoked.

20.1. Have you ever smoked **cigarettes?** Yes 

No 

*(“Yes,” means more than 20 packs of cigarettes in a lifetime or more than 1 cigarette each day for a year)*

*[if* ***yes****, ask questions 20A through 20D; otherwise, skip to Question 20.4)*

A. How old were you when you first started regular _____ _____ years old

cigarette smoking?

B. If you have stopped smoking, how old were you _____ _____ years old

when you last stopped? (If the participant has

not stopped smoking, record as code ‘999’.)

C. On average over the entire time that you i)____ ____ ____ cigarettes/day

smoke(d), about how many cigarettes per ii)___ ____ ____ cigarettes/week

day/per week do (did) you smoke?

D. On average over the entire time that you Manufactured 

smoke(d), do (did) you primarily smoke Hand-rolled 

manufactured or hand-rolled cigarettes?

20.4. Have you ever smoked **pipes of tobacco?** Yes ❑ No ❑

*(“Yes,” means more than 12 ounces of tobacco pipe in a lifetime)*

*[if* ***yes****, ask questions A through C; otherwise, skip to Question 20.5)*

A. How old were you when you first started regular _____ _____ years old

pipe smoking?

B. If you have stopped smoking, how old were you _____ _____ years old

when you last stopped? (If the participant has

not stopped smoking, record as code ‘999’.)

C. On average over the entire time that you i)____ ____ ____ grams/day

smoke(d), about how many pipes per ii)____ ____ ____grams/week

day/per week do (did) you smoke? (check MRC questionnaire)

20.5. Have you ever smoked **cigars, cheroots, or cigarillos?**  Yes ❑ No ❑

*(“Yes,” means more than 1 cigar / cheroots / cigarillos per week for one year at any time in your life)*

*[if* ***yes****, ask questions A through C; otherwise, skip to Question 20.7)*

A. How old were you when you first started regular _____ _____ years old

cigar/cheroot/cigarillo smoking?

B. If you have stopped smoking, how old were you _____ _____ years old

when you last stopped? (If the participant has

not stopped smoking, record as code ‘999’.)

C. On average over the entire time that you i) __ ___ ___ cigars etc/day

smoke(d), about how many cigars/cheroots/cigarillos per ii)__ __ __cigars etc/week

day/per week do (did) you smoke?

20.7. Have you ever smoked **cannabis?** Yes ❑

No ❑

(“Yes,” means more than 20 joints in a lifetime or more than 1 joint each month for a year at any time in your life)

*[if* ***yes****, ask questions A through C; otherwise, skip to Question 20.8)*

A. How old were you when you first started regular _____ _____ years old

cannabis smoking?

B. If you have stopped smoking, how old were you _____ _____ years old

when you last stopped? (If the participant has

not stopped smoking, record as code ‘999’.)

C. On average over the entire time that you i)___ ____ ___ joints/day

smoke(d), about how many cannabis joints per ii)___ ___ ___ joints/week

day/per week do (did) you smoke?

20.8. **Have you ever smoked or inhaled any other substance? (e.g. local, recreational smoked substances)**

Yes 

No 

20.8.1. specify type ___________________________________________

20.8.2. specify unit _________________________________e.g. pipes, joints

*[if* ***yes****, ask questions A through C; otherwise, skip to Question 24.1)*

A. How old were you when you first started regular __ __years old

(________) smoking?

B. If you have stopped smoking, how old were you _____ _____ years old

when you last stopped? (If the participant has

not stopped smoking, record as code ‘999’.)

C.On average over the entire time that you i)__ __ __ unit/day

smoke(d), about how many units ii)__ __ __unit/week

per day/per week do (did) you smoke?

*[If the participant currently smokes cigarettes (Question 20B is ‘999’), then ask Questions 21A and 21B. Otherwise, skip to Question 23]*

## 24.1. Not counting yourself, how many people in your household smoke

## regularly? __ __

24.2. Do people smoke regularly in the room where you work? Yes  No  Don’t work 

24.3. How many hours per day, are you exposed to other people's tobacco smoke

in the following locations?

24.3.1. At home ___ __hours

24.3.2. In workplace ___ __hour

24.3.3. Bars, restaurants, cinemas or similar social settings ___ __hours

24.3.4. Elsewhere ___ __hours

24.4. Did your father ever smoke regularly during your childhood? Yes 

No 

24.5. Did your mother ever smoke regularly during your childhood? Yes  No 

## Additional Co-morbidities

26. Has a doctor or other health care provider ever told you that you had:

26A. Heart disease Yes 

No 

26A.1 Heart failure Yes 

No 

26B. Hypertension Yes 

No 

26C. Diabetes Yes 

No 

26D. Lung cancer Yes 

No 

26E. Stroke Yes 

No 

26F. Tuberculosis Yes 

No 

*[If* ***yes*** *to 26F, then ask 26F1; otherwise, skip to Question 27]*

26F1. Are you currently taking medicine for tuberculosis? Yes 

No 

*[If* ***no*** *to 26F1, then ask 26F2; otherwise, skip to Question 27]*

26F2. Have you ever taken medicine for tuberculosis? Yes 

No 

27. Have you ever had an operation on your chest in which a part of Yes 

your lung was removed? No 

28. Were you hospitalized as a child for breathing problems prior to Yes 

the age of 10? No 

Don’t Know 

| **FORM COMPLETION** | | |
| --- | --- | --- |
| 29. | Date Form Completed |  |
| 30. | Initials of AIR Study Team Member |  |
