# Supplementary material for: Household air pollution, chronic respiratory disease and pneumonia in Malawian adults: A case-control study
Source: Wellcome Open Res. 2017 Oct 24;2:103. [Version 1] doi: 10.12688/wellcomeopenres.12621.1 (PMC5730861; doi:10.12688/wellcomeopenres.12621.1)
Supplement: Supplementary file 2 [file wellcomeopenres-2-13666-s0001.tgz › b08daa72-7ff2-42d9-aa60-863ece6c5113.docx]

# **Online Data Supplement**

**Household air pollution, chronic respiratory disease and pneumonia in Malawian adults: a case-control study**

Hannah R. Jary, Stephen Aston, Antonia Ho, Emanuele Giorgi, Newton Kalata, Mulinda Nyirenda, Jane Mallewa, Ingrid Peterson, Stephen B. Gordon, Kevin Mortimer

**Figure E1. Additional univariate analysis of exposures for HIV-positive and HIV-negative subgroups**

| **Exposures** | **HIV-positive subgroup** | | | | **HIV-negative subgroup** | | | |
| --- | --- | --- | --- | --- | --- | --- | --- | --- |
|  | **Cases**  **(n= 117)** | **Controls**  **(n= 169)** | **Unadjusted OR (95% CI)** | **p-value** | **Cases**  **(n= 28)** | **Controls**  **(n= 84)** | **Unadjusted OR (95% CI)** | **p-value** |
| **Participant characteristics** | | | | | | | | |
| Civil status^††^, n (%)  Married (reference)  Widowed  Divorced  Separated  Single | 68 (58.1)  11 (9.4)  14 (12.0)  7 (6.0)  17 (14.5) | 121 (71.6)  18 (10.7)  11 (6.5)  2 (1.2)  17 (10.1) | 1  1.09 (0.49-2.44)  2.26 (0.97-5.26)  **6.23 (1.25-30.83)**  1.78 (0.85-3.71) | --  0.839  0.058  **0.025**  0.124 | 20 (71.4)  2 (7.1)  3 (10.7)  0 (0)  3 (10.7 | 54 (64.3)  4 (4.8)  2 (2.4)  3 (3.6)  21 (25.0) | 1  1.35 (0.23-7.95)  4.05 (0.63-26.05)  1  **0.39 (0.22-0.62)** | --  0.740  0.141  --  **<0.001** |
| Current occupation^††^, n (%)  Paid employee (reference)  Paid domestic worker  Self-employed  Unemployed  Unpaid family worker  Student | 40 (34.2)  2 (1.7)  40 (34.2)  32 (27.4)  1 (0.9)  2 (1.7) | 47 (27.8)  2 (1.2)  37 (21.9)  78 (46.2)  0 (0)  5 (3.0) | 1  1.18 (0.16-8.72)  1.27 (0.69-2.35)  **0.48 (0.27-0.87)**  1  0.47 (0.09-2.56) | --  0.875  0.445  **0.015**  --  0.382 | 12 (42.9)  0 (0)  7 (25.0)  9 (32.1)  0 (0)  0 (0) | 29 (34.5)  3 (3.6)  16 (19.1)  30 (35.7)  0 (0)  6 (7.1) | 1  1  1.06 (0.27-1.98)  0.73 (0.27-1.98)  --  1 | --  --  0.922  0.530  --  -- |
| Education level^†††^, n (%)  Higher education (reference)  Secondary only  Primary only  None  Unknown | 7 (6.0)  45 (38.5)  59 (50.4)  6 (5.1)  0 (0) | 12 (7.1)  59 (35.1)  90 (53.8)  7 (4.2)  0 (0) | 1  1.31 (0.48-3.59)  1.12 (0.41-3.02)  1.47 (0.35-6.17)  -- | --  0.603  0.817  0.599  -- | 2 (7.1)  4 (14.3)  18 (64.3)  4 (14.3)  0 (0) | 12 (14.3)  33 (39.3)  33 (39.3)  5 (6.0)  1 (1.2) | 1  0.73 (0.12-4.50)  3.27 (0.66-16.26)  4.8 (0.65-35.20)  -- | --  0.732  0.147  0.123  -- |
| Carer for somebody with chronic illness^††^, n (%)  No (reference)  Yes | 77 (65.8)  40 (34.2) | 97 (57.4)  72 (42.6) | 1  0.70 (0.43-1.14) | --  0.137 | 22 (78.6)  6 (21.4) | 58 (69.1)  26 (31.0) | 1  0.61 (0.22-1.68) | --  0.337 |
| Cigarette smoking status, n (%)  Never smoked (reference)  Ex-smoker  Current smoker | 85 (72.7)  28 (23.9)  4 (3.4) | 124 (73.4)  26 (15.4)  19 (11.2) | 1  1.57 (0.86-2.87)  **0.31 (0.10-0.93)** | --  0.141  **0.038** | 14 (50.0)  10 (35.7)  4 (14.3) | 68 (81.0)  7 (8.3)  9 (10.7) | 1  **6.93 (2.25-21.36)**  2.16 (0.58-8.01) | --  **0.001**  0.250 |
| Passive tobacco smoke exposure^†††^, n (%)  No (reference)  Yes | 65 (55.6)  42 (44.4) | 90 (53.3)  79 (46.8) | 1  0.91 (0.57-1.47) | --  0.701 | 13 (46.4)  15 (53.6) | 54 (64.3)  30 (35.7) | 1  2.08 (0.87-4.94) | --  0.098 |
| **Household characteristics** | | | | | | | | |
| Roofing material^†††^, n (%)  Finished (reference)  Rudimentary  Natural | 112 (95.7)  0 (0)  5 (4.3) | 164 (97.0)  0 (0)  5 (3.0) | 1  --  1.46 (0.41-5.18) | --  --  0.554 | 25 (89.3)  0 (0)  3 (10.7) | 82 (97.6)  0 (0)  2 (2.4) | 1  --  4.92 (0.78-31.11) | --  --  0.090 |
| Wall material^††^, n (%)  Finished (reference)  Rudimentary  Natural | 79 (67.5)  34 (29.1)  4 (3.4) | 98 (58.0)  71 (42.0)  0 (0) | 1  **0.59 (0.35-0.98)**  1 | --  **0.043**  -- | 19 (67.9)  8 (28.6)  1 (3.6) | 57 (67.9)  26 (31.0)  1 (1.2) | 1  0.92 (0.36-2.38)  3.00 (0.18-50.33) | --  0.868  0.445 |
| Floor material^†††^, n (%)  Finished (reference)  Rudimentary  Natural | 93 (79.5)  0 (0)  24 (20.5) | 134 (79.3)  0 (0)  35 (20.7) | 1  --  0.99 (0.55-1.77) | --  --  0.968 | 20 (71.4)  0 (0)  8 (28.6) | 72 (85.7)  0 (0)  12 (14.3) | 1  --  2.40 (0.86-6.67) | --  --  0.093 |
| Number of people per room in household^†††^, median (IQR) | 1.5 (1.0-2.0) | 1.5 (1.0-2.0) | 1.09 (0.79-1.51)^‡^ | 0.604 | 1.7 (1.0-2.0) | 1.5 (1.0-2.0) | 1.15 (0.66-1.99) ^‡^ | 0.625 |
| Contact with children^†††^, n (%)  Does not live with children (reference)  Lives with children | 15 (12.8)  102 (87.2) | 27 (16.0)  142 (84.0) | 1  1.29 (0.65-2.55) | --  0.459 | 2 (7.1)  26 (92.9) | 14 (16.8)  70 (83.3) | 1  2.60 (0.55-12.23) | --  0.226 |
| Animal ownership^††^, n (%)  No animals (reference)  Owns animals | 86 (73.5)  31 (26.5) | 111 (65.7)  58 (34.3) | 1  0.69 (0.41-1.16) | --  0.161 | 13 (46.4)  15 (53.6) | 59 (70.2)  25 (29.8) | 1  **2.72 (1.13-6.55)** | --  **0.025** |
| Sleeps in same room as animals, n (%)  No (reference)  Yes | 117(100.0)  0 (0) | 167 (98.8)  2 (1.2) | 1  1 | --  -- | 27 (96.4)  1 (3.6) | 81 (96.4)  3 (3.6) | 1  1.00 (0.10-10.02) | --  1.000 |
| Population density (1000 people/ km^2^) ^†††^, median (IQR) | 4.2 (2.2- 8.0) | 4.2 (2.4-9.9) | 0.98 (0.920-1.02) ^‡^ | 0.256 | 3.9 (2.2-7.1) | 3.9 (2.2-9.9) | 0.96 (0.88-1.06) ^‡^ | 0.447 |
| **Pollution exposures** | | | | | | | | |
| Work related dust/smoke exposures^††^, n (%)  Never exposed (reference)  Previous exposure  Current exposure | 32 (27.4)  44 (37.6)  41 (35.0) | 33 (19.5)  66 (39.1)  70 (41.4) | 1  0.69 (0.37-1.28)  0.60 (0.32-1.12) | --  0.235  0.111 | 6 (21.4)  13 (46.4)  9 (32.1) | 32 (38.1)  23 (27.4)  29 (34.5) | 1  3.01 (1.00-9.11)  1.66 (0.52-5.22) | --  0.050  0.390 |
| Exhaled CO (ppm), median (IQR) | 4 (2-6) | 4 (3-6) | 0.98 (0.93-1.03) ^‡^ | 0.430 | 4 (3-6) | 3 (2-6) | 1.02 (0.96-1.10) ^‡^ | 0.479 |
| ^‡^ Per unit change. ^††^ *A priori* potential confounder with Likelihood Test Ratio p-value <0.2 therefore entered into the logistic regression model. ^†††^ *A priori* potential confounder with Likelihood Test Ratio p-value >0.2 therefore not entered into the logistic regression model.  IQR: interquartile range, ppm: parts per million | | | | | | | | |
