# Supplementary material for: Household air pollution, chronic respiratory disease and pneumonia in Malawian adults: A case-control study
Source: Wellcome Open Res. 2017 Oct 24;2:103. [Version 1] doi: 10.12688/wellcomeopenres.12621.1 (PMC5730861; doi:10.12688/wellcomeopenres.12621.1)
Supplement: Peer review history

# Peer review: Referee response for version 1

**DOI:** 10.21956/wellcomeopenres.13666.r28564

**Published:** 2017-12-13

**Authors:** Richard N. van Zyl-Smit

**Affiliations:** 1 Division of Pulmonology, Department of Medicine, UCT Lung Institute, University of Cape Town, Cape Town, South Africa

Indoor air pollution and pneumonia is a well-recognised association in children. The authors of this study evaluated indoor air pollution and the risk of pneumonia in adults. They showed no increased risk of pneumonia attributable to indoor air pollution exposure.

 This is a well conducted and very intensive study documenting pneumonia accurately (radiologically) and exposure (environmental monitoring) objectively. As with all studies there are limitations such as the case control design – although significant numbers were recruited, the evaluation of air pollution exposure was post event (pneumonia), and the number of confounders that need to be adjusted for.  What is striking is the absolute lack of effect OR 1 with 95% CI 0.99 – 1.01.

 It is unlikely that doing a prospective study with regular (pre event) air pollution monitoring, given the data presented would make any difference to the conclusion, and the short time between admission and subsequent air quality measurement precludes any real potential for significant differences in cooking behaviour before the event and after.

 What this study does highlight is the difference between children and adults – and that we can’t simply assume that the exposure and risks would be the same. Additionally the complex interactions of poverty, malnutrition, indoor air pollution, tobacco and alcohol require robust statistical methods. The univariate models showed effects of cooking with wood and odds of pneumonia to be 13. This was not evident in the HIV positive group – suggesting that HIV infection may play a far more significant role as a risk factor and the risk attributable to wood smoke does not significantly add to the high HIV risk. It is not possible by the nature of the study to evaluate the rate of pneumonia in the HIV negative vs. HIV positive groups for given exposure.

 It would be of value if the statistical community could evaluate such complex interactions/ confounders/ effect modifiers in such a cohort with newer tools, so that when we see a result such as this, which although plausible is a little unexpected, that we are certain that the lack of statistical effect is not due to ‘underpowered” statistical tools that can’t handle such complex and multifaceted interactions.

I have read this submission. I believe that I have an appropriate level of expertise to confirm that it is of an acceptable scientific standard.

# Peer review: Referee response for version 1

**DOI:** 10.21956/wellcomeopenres.13666.r27289

**Published:** 2017-11-20

**Authors:** Jennifer K. Quint

**Affiliations:** 1 Respiratory Epidemiology, Occupational Medicine and Public Health, Imperial College London, London, UK

This case control study set in Malawi compares hospitalised pneumonia patients and controls, with the aim of identifying potential risk factors for pneumonia. In addition, the results are stratified by HIV status allowing analysis as two separate case control studies. One of the strengths of this study is the confirmation of pneumonia with radiology in the setting of a large hospital in Blantyre. Controls were selected from enumeration areas nearby, and not from the hospital but frequency matched on age and gender to the cases. While this might not be the ideal methodology for finding controls, it is a common method in this environment. Inclusion and exclusion criteria were fairly strict meaning that study ineligibility on screening was relatively high. Air pollution monitoring was not always available with a delay from recruitment to monitoring start, which may contribute to the lack of association found between air pollution and pneumonia. Ideally exposure measurement would precede the outcome. Unsurprisingly, and in keeping with developed countries, there was an association between chronic respiratory disease and pneumonia. Working in this setting is never straightforward and the authors have certainly tried to undertake as robust a study as possible.

I have read this submission. I believe that I have an appropriate level of expertise to confirm that it is of an acceptable scientific standard.

# Response

**Published:** 2017-11-28

**Authors:** Hannah Jary

**Affiliations:** Liverpool School of Tropical Medicine, UK

Thank you for these encouraging comments, acknowledging some of the strengths of our study. We recognise that a limitation of the study is the measurement of air pollution exposure after the outcome of pneumonia. A cohort design would have avoided this issue with exposure classification, but would have required substantially more time and resources. In the context of a case-control design, we deliberately chose to delay the measurement of air pollution exposure in the cases (by 2-4 months), to allow time for the individual to recover to their normal ‘pre-pneumonia’ levels of function to try to obtain a more accurate assessment of their usual exposure.
